# Supplementary material for: Are carbon emissions trading and green financial instruments synergistic? -Comprehensive quantitative research based on content analysis
Source: PLoS One. 2024 Mar 7;19(3):e0298601. doi: 10.1371/journal.pone.0298601 (PMC10919601; doi:10.1371/journal.pone.0298601)
Supplement: S1 Data — (ZIP) [file pone.0298601.s001.zip › Data/地级市Z_F工作报告.zip!/║■▒▒/╩«╤▀╩╨/└┤╫╘╣1⁄2╓┌║┼í╛┬φ┐╦╩2╛▌═°í┐_2017.docx]

一、过去六年 工作回顾

　　（一）加快转型，综合实力显著提升。预计2016年实现地区生产总值1400亿元，年均增长9.1%；规上工业增加值510亿元，年均增长8.9%；全社会固定资产投资1400亿元，年均增长22.9%；地方一般公共预算收入首次突破百亿元大关，达100.2亿元，年均增长14.8%；社会消费品零售总额700亿元，年均增长14.8%，分别是2010年的1.9倍、1.4倍、3.4倍、2.3倍、2.3倍。三次产业结构由2010年的10.5:54.6:34.9调整为11.8:48.9:39.3。工业总产值突破2000亿元。接待国内外游客4700万人次，旅游总收入达360亿元，年均分别增长20.3%、24.7%，迈入全国百强旅游城市。创新驱动、质量兴市等战略深入推进，高新技术企业达180家，高新技术产业增加值占GDP比重由2010年的5.4%提高到13.2%。

　　（二）固本强基，农村面貌焕然一新。实施“四个百万”工程、“61产业强农计划”，全市粮食生产保持“十一”连增，特色基地达573万亩。新增农民专业合作社1794家、家庭农场527家、“三品一标”认证产品237个。农业增加值达165亿元，农产品加工产值达420亿元。实施丹江口库区移土培肥等项目86个，净增耕地8.4万亩。美丽乡村建设加快推进，完成生态、水利项目投资160亿元，解决安全饮水147.4万人；投入30亿元改造升级农村电网；新建县乡村道路9225公里。

　　（三）全域统筹，“一核多支点”格局加快形成。编制完成2049远景战略规划，修编城市总规、控制性详规等1162项，城乡规划实现全覆盖。新建城市干道30余条，维修市政道路190余条，改造背街小巷、微循环道路260余条。建成西气东输二线十堰支干线，天然气入户20.2万户。启动“海绵城市”建设，成功申创全国首批地下综合管廊试点城市。开发利用低丘缓坡，新增城镇建设用地6万亩。“两带”建设纵深推进，新增特色产业基地180万亩，建成环库公路382公里，城镇化率达58.4%。县域经济占全市经济比重由2010年的37.5%上升到42.9%。

　　（四）深化改革，发展动能不断增强。推进供给侧结构性改革，“放管服”改革持续深化，市级行政审批项目由879项减少到210项，取消行政事业性收费项目251个。开展“一枚公章管审批、一个部门管市场、一支队伍管执法”试点。推进商事制度改革，市场主体达27.4万户，年均增长18.4%。深化农村综合配套改革，建成三级土地流转平台、林权交易中心，整合使用涉农资金形成“郧阳模式”。成功引进5家股份制商业银行，82家企业挂牌上市。各项存款余额超过2000亿元，贷款余额超过1100亿元。招商引资1809亿元，累计出口29.1亿美元，利用外资10.9亿美元，年均分别增长29.4%、16.3%、25.2%。成立对口协作产业投资引导基金，实施项目233个，到资42.5亿元。

　　（五）内外兼修，生态文明建设纵深推进。统筹推进五城联创，成功创建国家卫生城市、国家森林城市和省级环保模范城市。建立绿色GDP考核机制，在全省率先实施环境保护“一票否决”、生态环境损害责任终身追究制。开展“向三大污染宣战”等专项活动，取缔“十小”企业329家，“五河治理”成为全国治污典范；实施“绿满十堰”行动，植树造林176.2万亩，森林覆盖率达64.7%，林地面积、森林面积、活立木蓄积量位居全省第一，建成国家和省级自然保护区、湿地公园29个。开展农村环境连片综合整治，创建国家和省级生态乡镇（村）101个。环境质量持续提升，空气优良天数达80%，地表水III类以上水质比例达91.2%，节能减排任务全面完成。

　　（六）以人为本，民生福祉持续改善。加大民生投入，民生及社会事业支出占地方一般公共预算支出比重达80%以上。城乡居民人均可支配收入分别达25980元、8556元，年均增长12.7%、16.1%。累计新增就业26.2万人，城镇登记失业率持续控制在4%以内。开展“四双”驻村帮扶，实施整村推进410个，搬迁贫困户5.2万户，减少贫困人口63.4万人。县域义务教育均衡发展通过国家认定，新改扩建中小学558所、幼儿园314所，新增校舍78万平方米。基本形成四级公共文化服务体系，社区文化活动室实现全覆盖。人口实现均衡发展，千人拥有病床7.85张，位居全省市州第一。低保补助标准增长1.33倍，实现应保尽保。五项社会保险综合参保率达90%，城乡医保参保率达99.2%，养老保险综合参保率达99%，基本建成覆盖城乡的社保体系。新增保障性住房2.63万套，改造棚户区14.67万户、农村危房6.3万户、老旧小区27个。各年度“十件实事”全面完成。

　　（七）依法治市，社会大局和谐稳定。扎实推进法治十堰建设，全面完成“六五”普法任务。自觉接受人大、政协监督，及时向人大报告工作、向政协通报情况，累计办理人大代表意见建议932件、代表走访选民建议712件，办结率100%，满意和基本满意率99.5%；办理政协提案2082件，办结率100%，满意和基本满意率99.8%。严格落实党风廉政建设主体责任，持续深化反腐倡廉，推进审计全覆盖。深入落实中央八项规定、省委六条意见，驰而不息纠正“四风”。扎实开展党的群众路线教育实践活动、“三严三实”专题教育和“两学一做”学习教育。健全完善社会治安防控、矛盾纠纷排查化解和安全生产监管常态化机制，群众安全感和治安满意度连续多年位居全省前列。深化精神文明创建，蝉联全国文明城市提名城市“两连冠”和省级文明城市“七连冠”。荣获全国双拥模范城“七连冠”。

　　二、今后五年政府工作的目标任务

　　今后五年政府工作的指导思想是：坚持以中国特色社会主义理论体系为指导，全面贯彻党的十八大和十八届三中、四中、五中、六中全会精神，深入贯彻习近平总书记系列重要讲话精神和治国理政新理念新思想新战略，按照“五位一体”总体布局和“四个全面”战略布局，围绕市第五次党代会提出的五大目标任务，深入实施“一城两带”、“外修生态、内修人文”战略，全面推进创新、协调、绿色、开放、共享发展，大力建设“五个十堰”，推进政府廉政建设，力争生态文明建设和转型跨越发展走在全省前列，为加快建设区域性中心城市而努力奋斗。

　　市第五次党代会提出了“决战脱贫攻坚、建设现代汽车城、建成全国生态文明示范区、建成全国文明城市、营造清明的政治生态”五大目标任务。根据上述目标任务，今后五年政府工作的总体目标是：地区生产总值年均增长9%左右，地方一般公共预算收入年均增长11%左右，城镇居民人均可支配收入年均增长9.5%以上，农村居民人均可支配收入年均增长11%以上。森林覆盖率达65%以上，空气质量优良率达到80%以上，丹江口水库水质稳定达到Ⅱ类标准，节能减排保持全省前列，生态文明示范区成为全国样板。城乡发展更趋协调，发展空间更加优化，“一核多支点”格局加快形成。民生福祉持续改善，公共服务体系更加健全，社会治理和谐有序，文明程度全面提高，人民群众获得感幸福感显著提升。

　　（一）决战脱贫攻坚，全面建成小康社会

　　——坚决打赢脱贫攻坚战。以脱贫攻坚统揽发展全局，变脱贫攻坚压力为加快农村发展机遇，紧盯“户脱贫、村出列、县摘帽”目标，创新“资金整合、利益联结、项目管理、考核评估、督查问责、精准退出”机制，推进产业扶贫、劳力转移、易地搬迁、医疗救助、扶智助学、兜底保障“六个一批”工程，到2018年全市83.6万建档立卡贫困人口全部脱贫销号、456个贫困村全部脱贫出列。2019年至2020年，巩固提高脱贫成果，贫困县全部脱贫“摘帽”。

　　——以脱贫攻坚促进农业发展。推进特色基地标准化、产品加工规模化、生产经营集约化，特色产业基地超过600万亩，农产品加工产值与农业总产值之比超过2:1。

　　——同步建成全面小康。到期末地区生产总值超过2000亿元，人均GDP超过1万美元，农村居民可支配收入超过13000元，城镇居民可支配收入超过38000元，城乡差距进一步缩小，人民幸福指数进一步提高，全市同步进入小康。

　　（二）建设现代汽车城，推进产业转型升级

　　——激发创新动能。实施“质量强市”和标准化战略，发扬“工匠精神”，培育精品名牌。深入实施“中国制造2025”、“互联网+”行动计划，促进产业发展迈向中高端。建设产业技术创新联盟、重点实验室等高水平创新平台，全社会研发投入占GDP比例达到2%。培育国家高新技术企业260家，增加值占GDP比重达到16%。

　　——加快建设现代汽车城。全力服务东风公司，积极支持东风商用车打造世界卡车第一“绿色”品牌，争取东风有限M9T发动机、东风小康经济型电动车项目落户十堰。巩固商用车全球行业领先地位，突破性发展专用车和新能源商用车。加快老工业基地调整改造，推进新型工业化示范基地建设，到期末规模以上工业总产值突破3000亿元，规模企业总数达1300家以上。

　　——构建一主多元产业体系。创新发展全域旅游，接待游客突破8000万人次，规模以上旅游企业500家以上。加快发展高效生态农业，推进茶叶、蔬菜、林果、草牧业、中药材、水产品等特色产业开发。培育壮大战略性新兴产业，重点发展高端装备制造、新能源、新材料、新技术等产业。做强做大电子商务、养生养老、健康服务、服务外包等现代服务业。到期末汽车产业产值突破2000亿，旅游总收入突破1000亿，农产品加工产值突破1000亿，服务业增加值突破1000亿，基本形成“一主四大四新”现代产业发展格局。

　　（三）统筹城乡发展，加快建设区域性中心城市

　　——优化提升中心城市功能。推进城区东拓、北扩、西进、南通、中优。加快主城区与郧阳区深度融合，形成滨江发展格局。改造老旧城区，转移和分流部分城市功能。加快地下综合管廊城市和“海绵城市”建设，有效治理行路难、停车难等各类“城市病”。改扩建武当山机场，打造区域性枢纽机场。加快构建以航空港、高速铁路、高速公路为主体的现代化交通体系，形成“三个两小时交通圈”。到期末十堰城区（含郧阳区）建成区面积达到150平方公里，人口承载能力达150万人，基本建成现代化山水园林城市。

　　——加快推进城乡一体化。加快汉江生态经济带建设，着力打造生态经济示范区、核心水源生态屏障区。加快京能热电、夹河关电站、孤山电站等重点项目建设，建成汉十高铁、十西高铁，推进丹西铁路、十宜铁路、十巫高速、十淅高速，丹江口、郧西、竹山、竹溪通用机场和丹江口库区航运等骨干工程建设。到期末全市常住人口城镇化率达60%，基本形成“三个1/3”人口承载格局。

　　——做强做大县域经济。支持各县市区竞进提质、特色发展，构建产业大对接、交通大联网、资源大共享格局。夯实县域产业支撑，打造一批百亿级园区、百亿级特色产业集群。

　　——建设美丽宜居乡村。紧扣“绿、净、齐、富、厚、和”，科学规划农田保护、村落分布、生态功能等空间布局，实施农村环境综合整治。建设一批生态家园示范村。实施新一轮农村公路、电网、宽带改造升级工程。

　　（四）深化改革开放，不断创优发展环境

　　——深化重点领域改革。持续推动政府职能转变，加大“放”的力度，强化“管”的能力，提升“服”的水平。推动“互联网+”政务服务，加快构建网上服务平台。推进相对集中行政许可权改革，组建行政审批局。推进行政审批标准化建设，逐步实现“一枚印章管审批、一个大厅管服务、一套机制管长效、一个标准管规范”。健全完善“四个清单”制度，重构审批流程，打造全省一流政务服务。加快推进“多证合一”、“证照分离”，建立公共资源交易平台。加强社会信用体系建设，创建“信用十堰”。

　　——推进供给侧结构性改革。深入推进“三去一降一补”。积极化解过剩产能，到2020年僵尸企业全部退出市场。有效化解房地产库存，放缓供地节奏，控制开发规模，优化用地结构。防范金融风险，落实差别化信贷政策。积极推广运用PPP模式，设立项目引导基金，组建金融控股公司，激活民间投资。清理规范中介机构，有效降低企业制度成本、税费负担、用工成本、能耗成本、物流成本和融资成本。深入推进农业供给侧结构性改革。加快补齐产业发展、基础设施、项目投资、脱贫攻坚、生态环保、县域经济等短板。

　　——全面提升开放合作水平。创新招商方式，提高招商质效，筹划组织新能源汽车、装备制造、健康养生等投资促进活动。加强“大流通、大开放、大通关”建设，推进国际贸易“单一窗口”建设。主动融入“一带一路”、长江经济带建设，深入对接湖北自贸区。抢抓首都功能区调整和产业转移机遇，与京津冀地区深入开展合作。加强秦巴山片区、丹江口库区及上游、鄂西圈等区域协作。

　　（五）加快绿色发展，建成国家生态文明示范区

　　——推进主体功能区建设。全面落实主体功能区建设方案，构建“一环三横三纵多点”生态安全屏障。创新生态文明体制机制，健全完善生态补偿和区域协调机制。实施山体保护条例和全民节能行动计划。推进工业园区循环化改造、传统制造业绿色化改造，建立城市废弃物资源化利用和无害化处理体系。将十堰经济技术开发区打造成为国家清洁生产示范区，办好“中国·丹江口水都论坛”、环库自行车大赛等活动。深化生态文明系列创建，到期末80%以上县市区、乡镇（街道）创成生态县、生态乡镇。

　　——加强生态建设与环境保护。严格执行环境保护“党政同责、一岗双责”、“一票否决”制度，落实最严格的耕地保护、水资源管理等制度。全面实施《丹江口库区及上游水污染防治和水土保持“十三五”规划》。开展大气、水、土壤污染防治行动，加强工业污染源、农业面源污染治理。加强水利基础设施建设，完善综合防洪减灾体系。加大“五河治理”和堵河水质保护力度，全面消除黑臭水体，确保入库河流水质稳定达标。深入开展“绿满十堰”行动，加强丹江口库区湿地保护，新建一批森林公园、湿地公园。深入推进“五城联创”，巩固提升国家卫生城市、国家森林城市创建成果，创建国家环保模范城市和国家生态城市。

　　（六）发展普惠民生，积极推进社会治理现代化

　　——全面发展社会事业。推进教育现代化，提高教育质量，保障教育公平，建设区域性教育强市。深化医药卫生体制改革，健全完善分级诊疗、现代医院管理、全民医保、药品供应保障、综合监管五项基本制度。推进“健康十堰”建设，基本建成秦巴山片区区域性医疗中心。加强公共体育设施建设，促进竞技体育发展。完善公共文化服务体系，保护和传承地方传统文化，加强汽车文化遗产保护，加快十堰大剧院、南水北调博物馆、中国车文化博物馆等项目建设，全面完成县级档案馆、图书馆、文化馆新建和改扩建任务。

　　——提高社会保障水平。完善就业服务体系，促进高校毕业生、农村劳动力、退役士兵等重点人群创业就业，做好淘汰落后产能中失业人员再就业工作。实施“全民参保计划”，全面推行社保“一卡通”。加强城乡救助体系建设，开展重大疾病救助，逐步提高救助标准。建设以居家为基础、社区为依托、机构为补充的多层次养老体系。加快棚户区和农村危房改造，加强保障性安居工程建设。

　　——创新社会治理。加强廉洁政府建设，形成良好政风，推动政府治理能力和治理体系现代化。加强行政监察和审计监督，构建有效防止腐败新机制。推进“七五”普法教育，深化法治十堰建设。推进城乡网格化管理，打通基层服务“最后一公里”。加强和改进信访工作，推进信访积案化解。深化平安十堰建设，完善治安防控体系，严厉打击违法犯罪活动。加强网络舆情监管，发展和规范网络空间。健全完善安全生产责任制，构建安全风险分级管控和隐患排查治理双重预防机制，坚决遏制重特大事故发生。加强食品药品安全监管，保障群众食品安全、用药安全、生命安全。

　　——建成全国文明城市。大力弘扬和践行社会主义核心价值观，切实加强社会公德、职业道德、家庭美德、未成年人思想道德建设，广泛开展群众性精神文明创建活动，深入推进“十星”创建全覆盖，整体提升社会文明程度和市民文明素质，加快建成全国文明城市。

　　三、扎实做好　2017年工作

　　今年发展的主要预期目标是：地区生产总值增长8.5%左右；规模以上工业增加值增长10%；全社会固定资产投资增长16%；社会消费品零售总额增长12%；地方一般公共预算收入增长9%；外贸出口增长8%；城乡居民收入增长高于经济增长；城镇登记失业率控制在4.5%以内；居民消费价格指数和节能减排控制在省定目标以内。围绕以上目标，重点做好七项工作：

　　（一）突出结构调整，促进提质增效

　　——加快工业转型升级。强化工业主导地位，加快构建现代产业新体系，实现规上工业增加值570亿元以上。一是做强做大主导产业。全力服务东风商用车中长期事业计划，积极支持东风公司十堰基地主辅分离，确保东风商用车动力总成变速箱一期、东风专用车设备科技公司新工厂投产见效。主攻“专、精、特、新、轻”，支持东风特商、东风小康、三环专汽、湖北大运等骨干企业发展，着力在消防、应急、物流、特种车等取得突破性进展。二是改造提升传统产业。加快纺织、建筑建材、冶金、化工等改造升级，优化存量、做强增量、量质双升，引导企业创新品种、提升品质、培育品牌。三是加快培植新兴产业。重点发展高端装备、智能制造、生物医药、新能源、新材料、电子信息等战略性新兴产业。四是扶持壮大中小微企业。着力振兴实体经济，防止资源、资金、资产“脱实向虚”。全面落实结构性减税、电价调整、降低社保费等政策，降低企业成本。

　　——大力发展现代农业。推进农业供给侧结构性改革，加快农业结构调整，促进农业增量增效。一是发展壮大特色产业。深入实施“61”产业强农计划，新建特色产业基地5万亩，改造中低产园15万亩。推进丹江口市、郧阳区、竹溪县、房县等省级农业产业化示范园区建设，支持丹江口市、竹溪县创建国家有机产品认证示范区。二是加快培育新型经营主体。新增合作社100家以上、家庭农场150家以上、专业大户1000家以上。三是加强农业基础设施建设。加快美丽乡村建设，推进农业面源污染、规模畜禽污染、网箱养殖等环境综合整治。加大投入力度，改造县乡道100公里，新改建通村公路1000公里。

　　——繁荣发展现代服务业。一是优化提升生态文化旅游业。完善南水北调源头生态文化旅游区、环郧阳湖旅游度假区功能，加快城郊生态文化游憩带建设，启动建设市游客集散中心、“东风1969”工业旅游区等重大项目。全市接待游客5400万人次、旅游收入415亿元以上，均增长15%。二是突破性发展现代物流业。启动华西农商城二期建设，加快三大物流集聚区、三大物流中心建设。优化农村物流布局设点，构建多层次物流网络。三是创新发展传统商贸业。推进“批发出城、零售进城”，支持商贸企业线上线下同步发展。四是加快发展新兴业态。重点发展电子商务、健康养老、金融保险、信息服务等服务业，促进消费升级。做优做强“淘宝·特色中国·十堰馆”等电商平台，支持竹溪建设“全国电子商务进农村综合示范县”。

　　——提高创新成果转化实效。强化创新驱动，新增高新技术企业20家，高新技术产业增加值200亿元、增长8%。推进秦巴山片区科技创新中心、国家科技成果转化服务示范基地、十堰中关村产业基地等建设。积极培养引进高科技人才，提升科技自主创新能力。完善“双创”支持政策，支持众创空间等“双创”平台建设，形成各类创新主体互促、草根与精英共创、线上与线下互动的局面。

　　（二）突出项目投资，厚植发展后劲

　　——强力推进重大项目建设。实施一批打基础、补短板、惠民生的重点项目，突出抓好16个省级、140个市级重点项目建设，完成全社会固定资产投资1600亿元以上。强化项目服务推进机制，对重点项目用地应保尽保。加快建设汉十高铁、京能热电、夹河关电站等重点项目，启动建设武西高铁十堰北站至武当山一级公路等项目，争取十西高铁、十巫高速、通用航空等重大项目达到开工条件。

　　——千方百计扩大有效投资。围绕产业发展、基础设施、社会民生等重点领域，策划论证一批大项目，力争更多项目挤进国家和省“笼子”。积极开展产业招商、主题招商、专业招商等活动，狠抓招商项目落地，确保新引进项目100个，力争达到120个。

　　（三）突出城乡统筹，优化区域格局

　　——做精做优中心城区。坚持新区与老城、生产与生活、形态与生态、地上与地下、硬件与软件并重，推进智慧城市建设，提升城市建管水平。以2049远景战略规划为引领，修编完成城市总体规划，统筹推进“多规合一”。新建三峡路、阳光大道二期、机场大道等道路。加快生态滨江新区建设，推进环郧阳湖、沧浪洲汉江大桥等项目建设。完成地下综合管廊试点项目建设，天然气入户2万户以上。加快城区园林绿化。推进数字城管，深入开展市容环境综合整治。

　　——扎实推进以人为核心的新型城镇化。统筹城乡建设布局、生产力布局和人口布局，促进城市均衡发展。纵深推进“两带”建设，重点推进6个县市区城区、25个国家和省级重点特色镇建设。加强基础设施建设。推进农村劳动力转移，城镇化率提高1个百分点。

　　——推动县域经济竞进提质。坚持“五化同步”，推进一二三产融合发展。积极争取全省县域经济发展调度资金，用好用足产业发展基金，支持各地发展壮大特色产业集群，力争县域GDP、县域工业增加值均增长9%以上。

　　（四）突出改革创新，释放发展动能

　　——持续深化改革。以供给侧结构性改革为主线，落实“三去一降一补”任务，推动供需结构有效匹配。深化“放管服”改革，健全完善清单管理模式，推进组建市行政审批局。深化生态文明体制改革，编制自然资源资产负债表，继续扩大领导干部自然资源资产离任审计。深化社会民生领域改革，推进机关事业单位养老保险、城乡居民医疗保险整合等改革。

　　——提高双向开放合作水平。坚持引进来和走出去并重、引进外资和对外投资并举，推动装备、技术、服务“走出去”。积极融入“一带一路”、长江经济带、湖北自贸区建设，推进汉江生态经济带、秦巴山片区等区域协作。积极争取国家部委对口援助协作，抓好对口援疆工作。

　　（五）突出生态文明，提升环境质量

　　——优化生态空间。实施国土空间开发优化工程，严控城市边线，守牢生态红线，扎紧耕地底线，完成全域永久基本农田划定。继续推进国家生态文明先行示范区三年行动计划。加强石漠化综合治理、水土保持、小流域治理等生态工程建设，抓好废弃厂（矿）区环境治理。完成植树造林20万亩、义务植树1000万株以上。

　　——加强生态建设与环境保护。贯彻落实国家《“十三五”生态环境保护规划》，深入推进国家环保模范城和生态市创建。狠抓突出环境问题整治，加大违法行为查处力度。强化全流域“河长制”管理，推进“五河治理”项目建设。大力推进节能减排，实施煤改气等工程，加强重点单位污染排放监测。抓好固体废物、扬尘、餐饮油烟等污染治理，全面淘汰“黄标车”，城区空气质量优良天数保持在80%以上。

　　（六）突出民生保障，增进社会和谐

　　——加大脱贫攻坚力度。强化产业扶贫，推进56个国家旅游扶贫试点村建设，培育示范企业100家。推进易地扶贫搬迁，完成5万户15万人搬迁任务。推广郧西县国家贫困劳动力输出对接试点经验，培训贫困劳动力3万人次，转移就业1万人。落实兜底保障等政策，实现贫困人口全覆盖。深化“四双”驻村帮扶，市直单位帮扶重点村再出列30%。完成16.5万贫困人口、203个贫困村脱贫出列任务，贫困户人均可支配收入增长15%以上。

　　——切实加强社会保障。推进更高质量的稳定就业，新增就业3.6万人，再就业1.8万人。完善社会救助标准量化调整机制，确保城乡低保水平与经济社会发展同步。积极开展特大疾病医疗等社会救助，织牢托底安全网。巩固五项社会保险成果，综合参保率保持在90%以上。加强养老设施建设，探索推进医养融合新模式，新增养老机构10家以上，养老床位增长15%以上。推进保障性安居工程建设，规范公租房管理，加大棚户区改造货币化安置力度。

　　——加快发展社会事业。推进教育公平，协调发展各类教育。加快车城高中新校区等建设，建成市高级职业学校、柳林中学新校区、十堰外国语学校。加快推进各县市“两馆两场”建设，提升免费开放服务水平。推进“健康十堰”建设，为群众提供全方位、全周期健康服务。深化医药卫生体制改革，推进城市公立医院改革，建立健全分级诊疗、药品供应保障等制度。推进基本生育服务，落实“全面二孩”政策，综合治理出生人口性别比不平衡问题。

　　——提高社会治理水平。全面推进依法治市，深入开展“七五”普法。完善重大决策社会稳定风险评估、社会矛盾排查预警等机制，提升突发公共事件应急处置能力。持续抓好信访维稳工作。加强社会信用体系建设。深化“平安十堰”建设。强化落实安全生产责任制。加强食品药品监管。抓好全国文明城市等测评工作。加强国防动员建设。

　　（七）突出自身建设，推进治理体系现代化

　　——严守规矩，依法履职。坚持纪严于法、纪在法前，切实增强政治意识、大局意识、核心意识、看齐意识。深入实施《法治政府建设实施纲要（2015-2020）》，加快建设“职能科学、权责法定、执法严明、公开公正、廉洁高效、守法诚信”的法治政府。切实抓好地方立法，完善依法科学民主决策机制。深化权力制约监督，严格实行重大决策终身追究制、责任倒查制和行政执法自由裁量权基准制。依法接受人大及其常委会的监督，自觉接受人民政协的民主监督，主动接受社会监督和舆论监督。积极听取民主党派、工商联、无党派人士和人民团体意见建议。认真办理人大议案、建议和政协提案。加强政务公开，让权力在阳光下运行。

　　——敢于担当，勤勉履职。牢记宗旨意识，恪尽为民职守，以“第一力度”狠抓“第一要务”。以功成不必在我、功成定必有我的境界，以“钉钉子”精神脚踏实地推进工作，敢担重任，敢于碰硬，勤勉敬业，善做善成。严格执行工作目标考核责任制，健全完善督查问责机制，坚决整肃庸政懒政怠政行为，切实解决不敢为、不会为、乱作为等问题。旗帜鲜明地鼓励担当者、保护改革者、宽容失误者，让广大干部愿干事、敢干事、干成事。

　　——强化监督，廉洁履职。严格落实《关于新形势下党内政治生活的若干准则》、《中国共产党党内监督条例》和《湖北省行政问责办法》。认真履行“一岗双责”，全面落实党风廉政建设主体责任，履行政府党组管党治党责任。严格执行中央八项规定、省委六条意见，重拳整治各种顶风违纪行为，重手解决群众身边的不正之风和腐败问题。强化行政监察和审计监督，规范政府权力运行。厉行勤俭节约，反对铺张浪费，严防“四风”反弹，严控“三公”支出，切实做到为民、务实、清廉。
